# Unraveling the parameters and biological mechanisms of CO_2_ laser therapy for acute pain relief

**DOI:** 10.3389/fneur.2023.1271655

**Published:** 2023-10-20

**Authors:** Xianggang Wang, Jiaqi Liu, Zhonghan Wang, Chunming Guo, Hongjia Lan, Shibin Feng, He Liu, Xun Gao, Dongming Zhang, Lintao Zhu, Hui Jin, Jincheng Wang

**Affiliations:** ^1^Orthopaedic Medical Center, The Second Hospital of Jilin University, Changchun, China; ^2^Orthopaedic Research Institute of Jilin Province, Changchun, China; ^3^School of Physics, Changchun University of Science and Technology, Changchun, China; ^4^Academy of Chinese Medical Sciences of Jilin Province, Changchun, China; ^5^Changchun Ideal Medical Technology Co., Ltd., Changchun, China

**Keywords:** CO_2_ laser, acute pain relief, analgesic effect, opioid receptor, formalin test

## Abstract

Acute pain-related pathology is a significant challenge in clinical practice, and the limitations of traditional pain-relief drugs have made it necessary to explore alternative approaches. Photobiomodulation (PBM) therapy using CO_2_ laser has emerged as a promising option. In this study, we aimed to identify the optimal parameters of CO_2_ laser irradiation for acute pain relief through *in vivo* and *in vitro* experiments. First, we validated the laser intensity used in this study through bone marrow mesenchymal stem cells (BMSCs) experiments to ensure it will not adversely affect stem cell viability and morphology. Then we conducted a detailed evaluation of the duty cycle and frequency of CO_2_ laser by the hot plate and formalin test. Results showed a duty cycle of 3% and a frequency of 25 kHz produced the best outcomes. Additionally, we investigated the potential mechanisms underlying the effects of CO_2_ laser by immunohistochemical staining, and found evidence to suggest that the opioid receptor may be involved in its analgesic effect. In conclusion, this study provides insights into the optimal parameters and underlying mechanisms of CO_2_ laser therapy for effective pain relief, thereby paving the way for future clinical applications.

## 1. Introduction

Pain is a significant global health concern, affecting a large proportion of the adult population worldwide. It has been reported that 20% of adults experience frequent pain, and 10% are diagnosed with chronic pain annually (1). Pain is an afflictive experience that is characterized as “An unpleasant sensory and emotional experience associated with, or resembling that associated with, actual or potential tissue damage” (2). While opioids have long been considered frontline analgesics, their utility is limited due to undesirable side effects such as respiratory depression and abuse liability (3). Therefore, there remains a pressing need to identify alternative, less harmful approaches to pain relief.

Photobiomodulation (PBM), also referred to as low-level laser therapy (LLLT), has shown great potential in relieving neurogenic pain (4, 5). This treatment method uses low-power laser to promote wound healing, reduce inflammation, prevent tissue death, and alleviate neurogenic pain (6). Among various types of PBM lasers, the CO_2_ laser has been found to be particularly effective in pain relief. Originally used in surgery for ablation and tissue section, the CO_2_ laser has been found to reduce post-operative analgesics and pain (7). Although low-level CO_2_ laser therapy has demonstrated a superior effect for acute pain relief, the underlying mechanism is still unclear. Some researchers hypothesize that the pain-relieving effects may be due to the reversible blockage of nociceptive signals in afferent neurons, especially those related to nociceptive stimuli transmission, such as Aδ and C fibers (8). Others propose that the therapy may impact proteins involved in nociceptive transmission by increasing the synthesis and release of β-endorphin or suppressing the release of substance P (9). While opinions vary, a consensus has not yet been reached, and further research is required for a more in-depth exploration of the subject.

Furthermore, the terminology of “low level laser therapy” and “non-ablative CO_2_ laser therapy” has been replaced with “photobiomodulation” since 2016, as there was no clear definition for what “low level” meant (6). However, the protocol for CO_2_ laser therapy is still undefined, and standard precautions to protect patients must be considered before laser irradiation. This includes positioning the patient correctly, using non-analgesic hydrogel on the lesion, and providing eye shields to protect against laser impact (10). The optimal parameters for CO_2_ laser therapy, such as power, distance from skin, and time of scanning during the continuous mode, have yet to be clearly defined and require further investigation.

The efficacy and optimal parameters of CO_2_ laser therapy for acute pain remain uncertain. In this study, we aimed to identify the most suitable parameters for pain relief by using a sophisticated CO_2_ laser instrument that enables accurate adjustment of various parameters. To this end, we employed classic pain models such as the hot plate test and formalin test to evaluate pain intensity under different CO_2_ laser parameters. Additionally, we investigated the underlying mechanisms of CO_2_ laser therapy by testing several pain-related proteins. This study aimed to elucidate the optimal parameters and mechanisms of CO_2_ laser therapy for the effective relief of acute pain.

## 2. Materials and methods

### 2.1. Materials

All the ultra-pure water used in the experiments was purified by a Mili-Q A10 filtration system (Milipore, Billerica, MA, United States). Phosphate-buffered saline (PBS) was obtained from Solarbio (Beijing, China). Low glucose Dulbecco’s Modified Eagle’s Medium (LGDMEM), fetal bovine serum (FBS), and streptomycin double antibody were obtained from Gibco (Grand Island, NY, United States). Hematoxylin and Eosin (H&E) stain dye solution was purchased from Thermo Fisher Scientific Co., Ltd. (Shanghai, China). Calcein-AM-PI staining kit were obtained from Bestbio (Nanjing China). Rhodamine-phalloidin was obtained from Solarbio (Beijing, China). Opioid receptor antibody, the voltage-gated Na (+) channel subtype Nav1.7 antibody, and the P substance receptor antibody were purchased from Abcam (MA, United States). Goat antirabbit IgG were purchased from the Life Technologies (United States).

### 2.2. Stem cell viability and morphology evaluation

In this study, we aimed to investigate the effects and mechanisms of CO_2_ laser with different duty cycle and frequencies on alleviating acute pain in rats. In addition to the parameters of interest, the following CO_2_ laser emission-related parameters were as follows: a wavelength of 10.6 μm, a repetition frequency of 1–100 kHz, a radiant flux density ranging from 5.5 to 250.5 mW per square centimeter, and a continuous pulse duration between 0.2 and 19.2 ms, laser out maximum power of 30 W, laser beam diameter of 1.8 ± 0.2 mm. To explore the effects of CO_2_ laser irradiation on cells, we conducted an *in vitro* assessment of the viability and morphology of rabbit bone marrow mesenchymal stem cells (BMSCs). BMSCs were obtained from healthy 1 week-old New Zealand rabbits and were cultured in LG-DMEM medium supplemented with 10% PBS and 1% streptomycin-penicillin in a humidified incubator at 5% CO_2_ and 37°C. The culture medium was replenished every 2–3 days as previously reported (11). The third generation was applied to evaluate the cell viability and morphology. The experiment was divided into four groups, the control groups without CO_2_ laser irradiation, the 20 kHz group, the 25 kHz group, and the 80 Hz group. The duty cycle of all the irradiated groups was 3%. The time of irradiation was set up 10 min every day. Briefly, the BMSCs were seeded at the density of 2 × 10^4^ cells per well in 24-well plates. With or without irradiation, after cultured for 3 days, the cell viability test was carried out according to the manufacturer’s protocol of the Calcein-AM/PI Double Stain Kit. The cell viability of BMSCs was evaluated by a confocal laser scanning microscope (CLSM, FV1000, Olymps, Japan). Quantitative analysis was conducted using Image J software (NIH, Bethesda, MD, United States). Ten random images were selected from each sample for quantitative analysis to calculate the proportion of viable cells in each group.

As for the morphological assessment of the BMSCs, the cytoskeleton of F-actin was stained by rhodamine-phalloidin. BMSCs were seeded in 12-well plates at a density of 1 × 10^4^ cells per well. Following irradiation with CO_2_ laser or without for 3 days, the cells were fixed with 4.0% paraformaldehyde for 10 min and permeabilized with Triton X-100 for 5 min. Finally, the samples were stained with Rhodamine-phalloidin in the dark for 30 min and re-stained with DAPI for 5 min. The confocal laser scanning microscope was employed to capture the images and quantitative analysis was conducted using Image J software.

### 2.3. Animals

All procedures were conducted in compliance with the National Institutes of Health’s Guide for the Care and Use of Laboratory Animals. The animal experimental protocols were approved by the Animal Care and Use Ethics Committee of Jilin University. Male Sprague-Dawley (SD) rats (8 weeks-old; 220–250 g; the Animal Center, College of Basic Medical Science, Jilin University) were used in all experiments. The rats were maintained in a controlled environment with a 12 h light/dark cycle, constant temperature of 25°C, and a relative humidity of 50–70%. They were given free access to food and water. Prior to the experiments, all animals were allowed to acclimate to the laboratory environment for a period of 7 days.

### 2.4. The hot plate tests

The hot plate test was employed to assess the potential analgesic effect of CO_2_ laser irradiation on thermal hyperalgesia. To investigate the effect of different duty cycles and frequencies of CO_2_ laser, a total of 36 rats were employed in the hot plate experiments. In the duty cycles section, the animals were divided into five groups, each consisting of six animals. The duty cycles of the laser irradiation were categorized into five groups: 1, 3, 5, 7, and 10%. The CO_2_ laser was positioned at a 30-degree angle beneath the rats and was directly applied to the plantar surface of their hind paws while they were housed in cages. The distance between the rats and the laser probe was maintained at 40 ± 5 cm. Prior to the hot-plate test, the rats underwent a 10 min irradiation session with a defined duty cycle. The test was conducted by placing the rats individually on an electronic hot-plate that was maintained at a temperature of 55 ± 1°C. The reaction time was measured from the time the rats were placed on the hot-plate until a latency response of hind-paw jump or lick was recorded. A cutoff time of 30 s was set to prevent tissue damage. Each animal was tested three times, with a 10 min interval between each measurement, and the average value of reaction time was calculated for analysis. Reaction time was measured both before and after CO_2_ laser irradiation, with the elongation ratio used to determine pain relief. The remission rate (*R*) was calculated using the formula *R* = T2/T1 × 100%, where *R* represents the remission rate, T1 represents the reaction time before irradiation, and T2 represents the reaction time after irradiation.

To investigate the impact of different frequencies of CO_2_ laser on relieving thermal hyperalgesia, rats were assigned into six groups with six animals in each group. The groups included a control group without any laser irradiation, and irradiation groups with frequencies of 20 kHz, 35 kHz, 50 kHz, 65 kHz, and 80 kHz. The remission rate in each group was assessed using the same method described previously. The measurements were repeated three times for each animal with a 10 min interval between each measurement, and the average value of reaction time was used for analysis.

### 2.5. The formalin tests

To evaluate the potential analgesic effect of CO_2_ laser at different frequencies, a smaller-scale study was conducted using a formalin test in rats. An additional total of 42 rats were used for the study. The animals were randomly allocated to one of seven groups, with each group comprising six rats. The groups included a control group that received no laser irradiation, and six irradiation groups that were exposed to frequencies of 20 kHz, 25 kHz, 30 kHz, 35 kHz, 40 kHz, and 45 kHz, respectively. Following the injection of 100 μL of 5% formalin into the right hind paw of each animal, CO_2_ laser irradiation was applied for 10 min. When the CO_2_ laser irradiation was completed, the rats’ behavioral responses were recorded using a camera for a duration of 50 min. The duration of time during which the rats licked and flinched their right hind paw was recorded every 5 min. The total time of licking and the number of flinches were calculated at the end of the experiment. After 6 h of the formalin test, the rats were euthanized using an overdose of analgesic injection. The right hind paws of the rats were collected for histological testing.

### 2.6. The histology and immunohistochemical staining

In order to investigate the expression of nociception-related genes in the irradiated area, we performed double immunofluorescent staining of the opioid receptor, voltage-gated Na (+) channel subtype Nav1.7 protein, and P substance receptor. Briefly, the samples were decalcified and embedded in paraffin before sectioning to a thickness of 5 μm. Tissue sections were incubated with the primary antibodies overnight at 4°C, followed by three washes with PBS. Subsequently, the tissue sections were incubated with goat anti-mouse immunoglobulin G conjugated to fluorescein isothiocyanate as the secondary antibody for 5 h. The intensity of the three nociception-related proteins was visualized using an optical microscope, and quantitative analysis was performed using Image J software.

### 2.7. Statistical analysis

All the data in experiments were shown as the mean ± standard deviation (SD). The Shapiro–Wilk tests were conducted to assess the normality of our datasets. Statistical analyses were performed exclusively on datasets that conformed to normal distribution. The statistical analyses were carried out using one-way analysis of variance (ANOVA) with Tukey’s *post hoc* analysis to test the variation between groups. All the statistical analyses were performed by SPSS 19.0 software (SPSS Inc., Chicago, United States). *p* < 0.05 was set as a statistically significant difference (*p < 0.05, **p < 0.01, and ***p < 0.001). All experiments were repeated independently at least three times.

## 3. Results

### 3.1. The cytotoxicity of the CO_2_ laser

**Figure 1:**
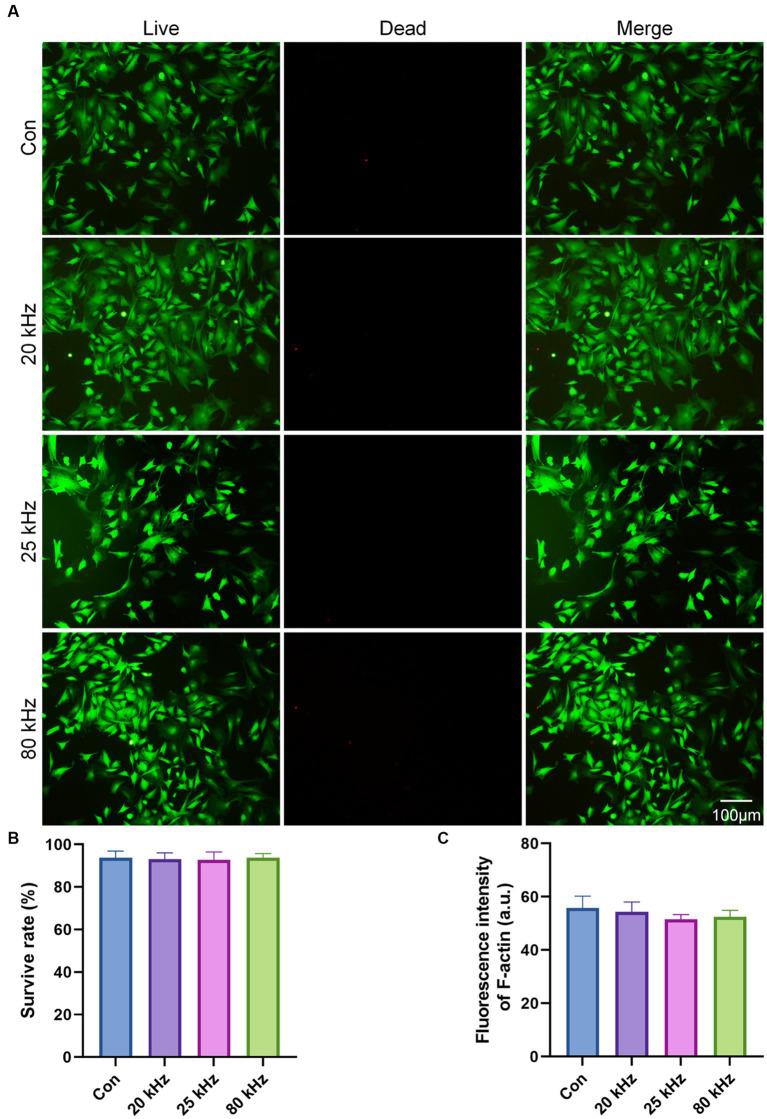
The cytotoxicity of the CO_2_ laser. **(A)** Calcein AM/PI staining of the live (green) and dead (red) cells (*n* = 3). **(B)** Quantitative analysis of cell survival rate according to Calcein AM/PI staining (*n* = 3). **(C)** Quantitative analysis of F-actin fluorescence intensity (*n* = 3). The data are expressed as the mean ± SD. Statistical analysis was conducted using one-way ANOVA followed by Tukey’s *post hoc* test for multiple comparisons. *p < 0.05, **p < 0.01, and ***p < 0.001.

BMSCs are commonly used in bone rehabilitation pain departments due to their therapeutic potential. Therefore, in this study, we chose to investigate the impact of CO_2_ laser irradiation on the viability and morphology of BMSCs. The cell viability results are illustrated in Figure 1A. Our findings show that there were no significant differences among the four groups after a 3 days irradiation period. The survival rates of the control group were 93.60 ± 3.21%, of 20 kHz were 93.10 ± 3.00%, of 25 kHz were 92.67 ± 3.78%, and of 80 kHz were 93.12 ± 2.08%, as shown in Figure 1B (*p* > 0.05). Notably, compared to the control group without CO_2_ laser irradiation, the viability of BMSCs was similar after irradiation with various frequencies for three days in the irradiated groups. Thus, the given parameters of CO_2_ laser did not exhibit any cytotoxic effects on BMSCs. Moreover, no thermal effects were observed during the irradiation process.

**Figure 2:**
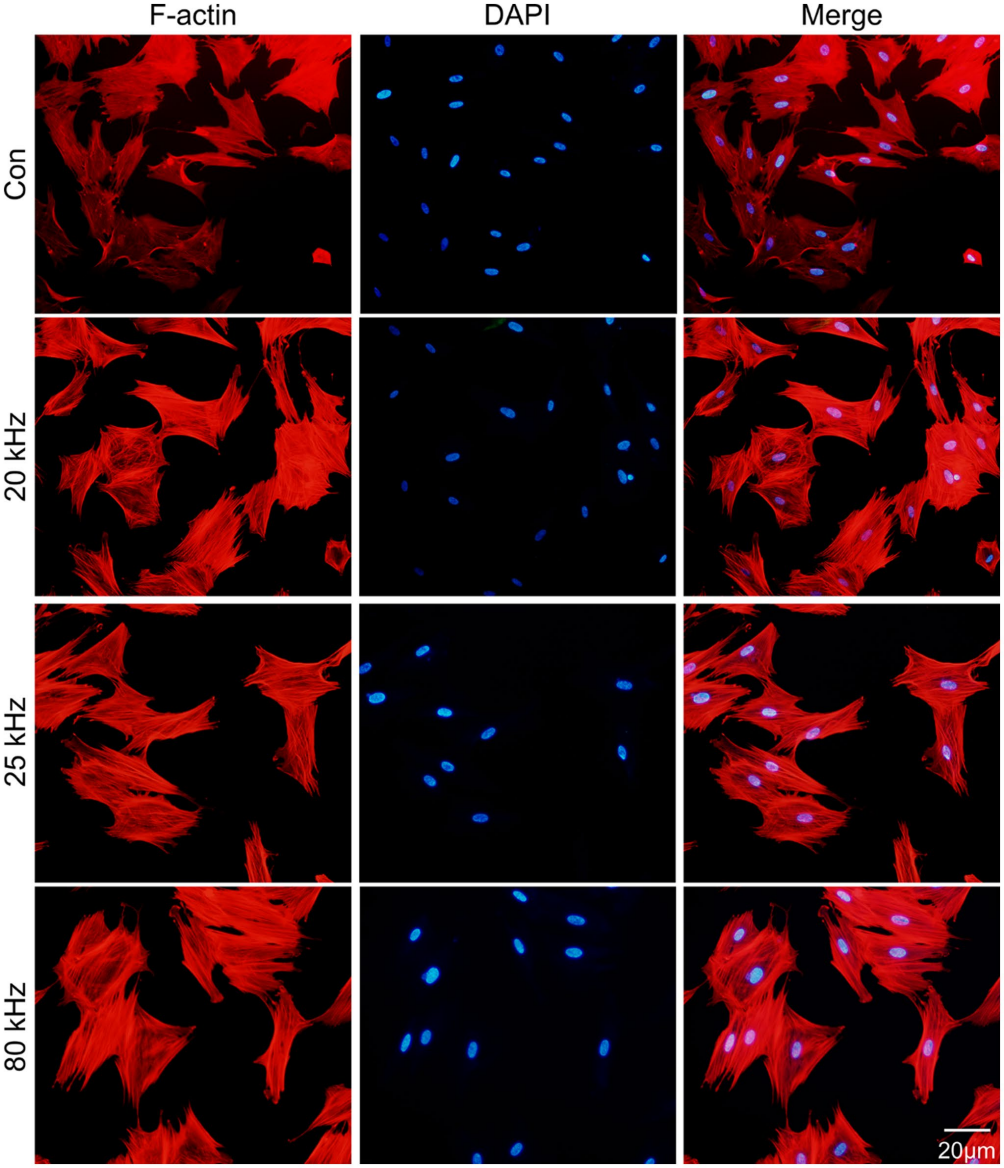
Fluorescent images of the rhodamine marked F-actin (red) and DAPI (blue) staining (*n* = 3).

As for the effect of CO_2_ laser on BMSCs morphology, the F-actin staining results are presented in Figures 1C, 2. The organization of F-actin filaments in BMSCs was assessed to further investigate the effects of CO_2_ laser irradiation on cell morphology. As shown in Figure 1C, no significant differences in the organization of F-actin filaments were detected among the four groups (*p* > 0.05). In Figure 2, the control group exhibited well-organized F-actin filaments after 3 days of incubation. Similar results were observed in the three irradiated groups, with BMSCs showing a uniformly distributed F-actin filament morphology post-irradiation. Thus, the present study suggests that the given parameters of CO_2_ laser are unlikely to impact the cytoskeletal organization of BMSCs, indicating the safety of clinical use.

### 3.2. The suitable parameter of the duty cycle

**Figure 3:**
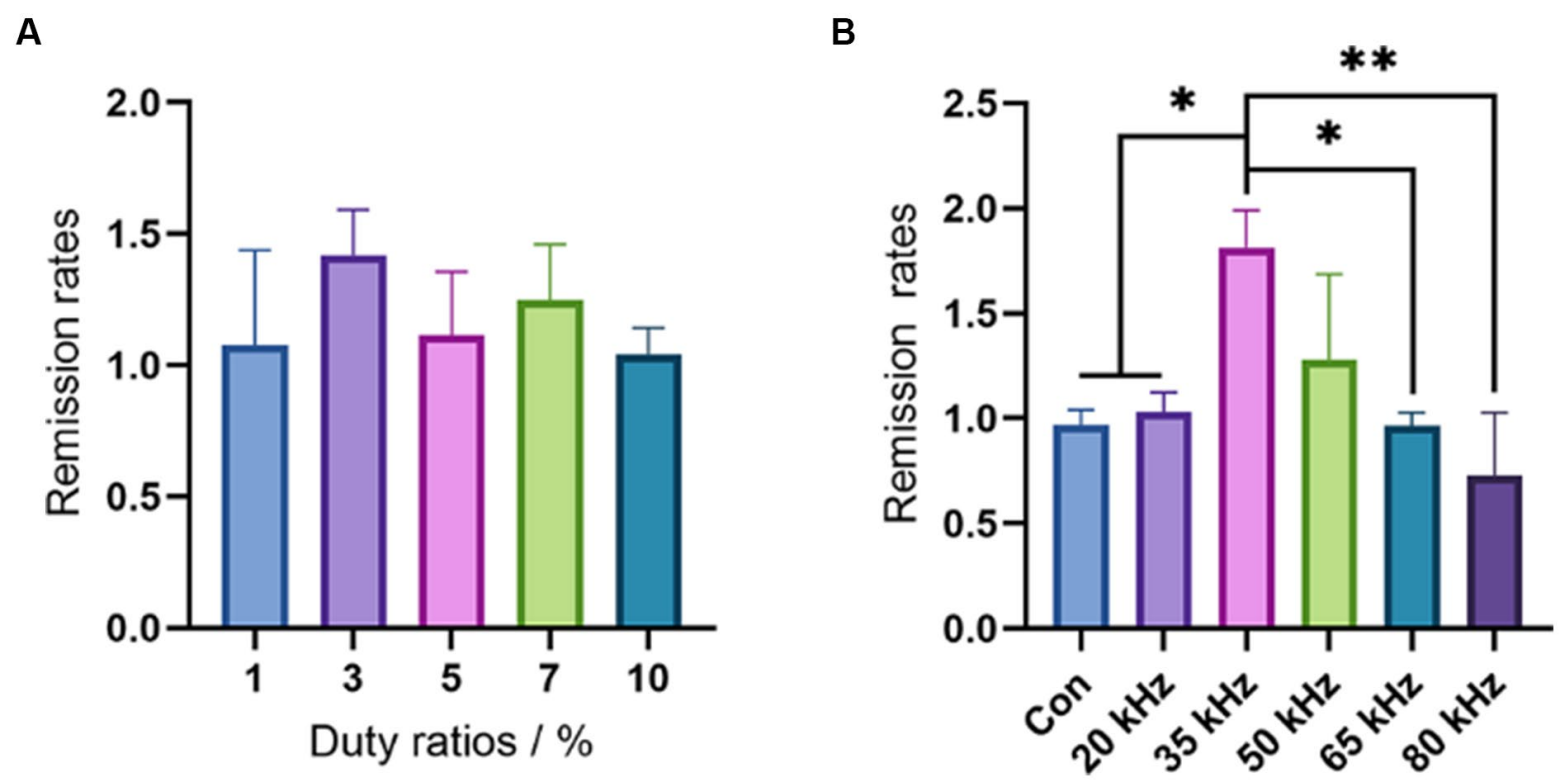
Results of the hot plate test. **(A)** The duty cycles selection by the hot plate tests (*n* = 6). **(B)** The frequency effects of CO_2_ laser in a wide range (*n* = 6). The data are expressed as the mean ± SD. Statistical analysis was conducted using one-way ANOVA followed by Tukey’s *post hoc* test for multiple comparisons. *p < 0.05, **p < 0.01, and ***p < 0.001.

In this study, we investigated the influence of duty cycle, which represents the exact ratio of CO_2_ laser exposure, on the thermal hyperalgesia relief effect of CO_2_ laser irradiation. As the CO_2_ laser is pulsed working, a larger duty cycle results in a stronger laser irradiation intensity. To avoid any thermal effect, we selected a low level of duty cycles ranging from 1 to 10% and evaluated the effect of CO_2_ laser under different duty cycles using the hot plate test. There was no increase in skin temperature within this duty cycle interval. As shown in Figure 3A, the pain relief effect was found to improve from 1 to 3%, with a subsequent trend of decline from 3 to 10%. Although there were no statistical differences among the different duty cycles (*p* > 0.05), the single peak curve suggested that a duty cycle of 3% may be the optimal choice for pain relief. Thus, in the subsequent experiments, we used CO_2_ laser irradiation under a duty cycle of 3%. However, due to the limited number of animals and wide deviation in the behavioral measures, further studies with larger sample sizes are required to verify the optimal duty cycle for pain relief.

### 3.3. The suitable parameter of the frequency

After selecting the optimal duty cycle of 3%, we evaluated the effect of different frequencies on pain relief using the hot plate test in a range from 20 kHz to 80 kHz. As shown in Figure 3B, there were significant differences in pain-relieving effects among the different frequencies. None of the frequencies resulted in any thermal effects. Notably, the frequency of 35 kHz showed a significant improvement in pain relief compared to the control (*p* < 0.05), 20 kHz (*p* < 0.05), 65 kHz (*p* < 0.05), and 80 kHz (*p* < 0.01), while 50 kHz exhibited a better remission rate than the control group, although no significant difference was observed (*p* > 0.05). In contrast, CO_2_ laser irradiation under frequencies of 20 kHz, 65 kHz, and 80 kHz showed no difference compared with the control group (*p* > 0.05). Intriguingly, the frequency of 80 kHz even had an aggravating effect on pain. These results highlight the crucial role of CO_2_ laser frequency in pain relief, with the outcome being highly sensitive to changes in frequency. Although 35 kHz exhibited the best result among the wide frequency range of 20 kHz to 80 kHz, further investigation is necessary to determine the most appropriate frequency. Thus, we plan to evaluate the pain relief effect of CO_2_ laser therapy under a narrow interval of different frequencies ranging from 20 kHz to 50 kHz in our subsequent experiments.

**Figure 4:**
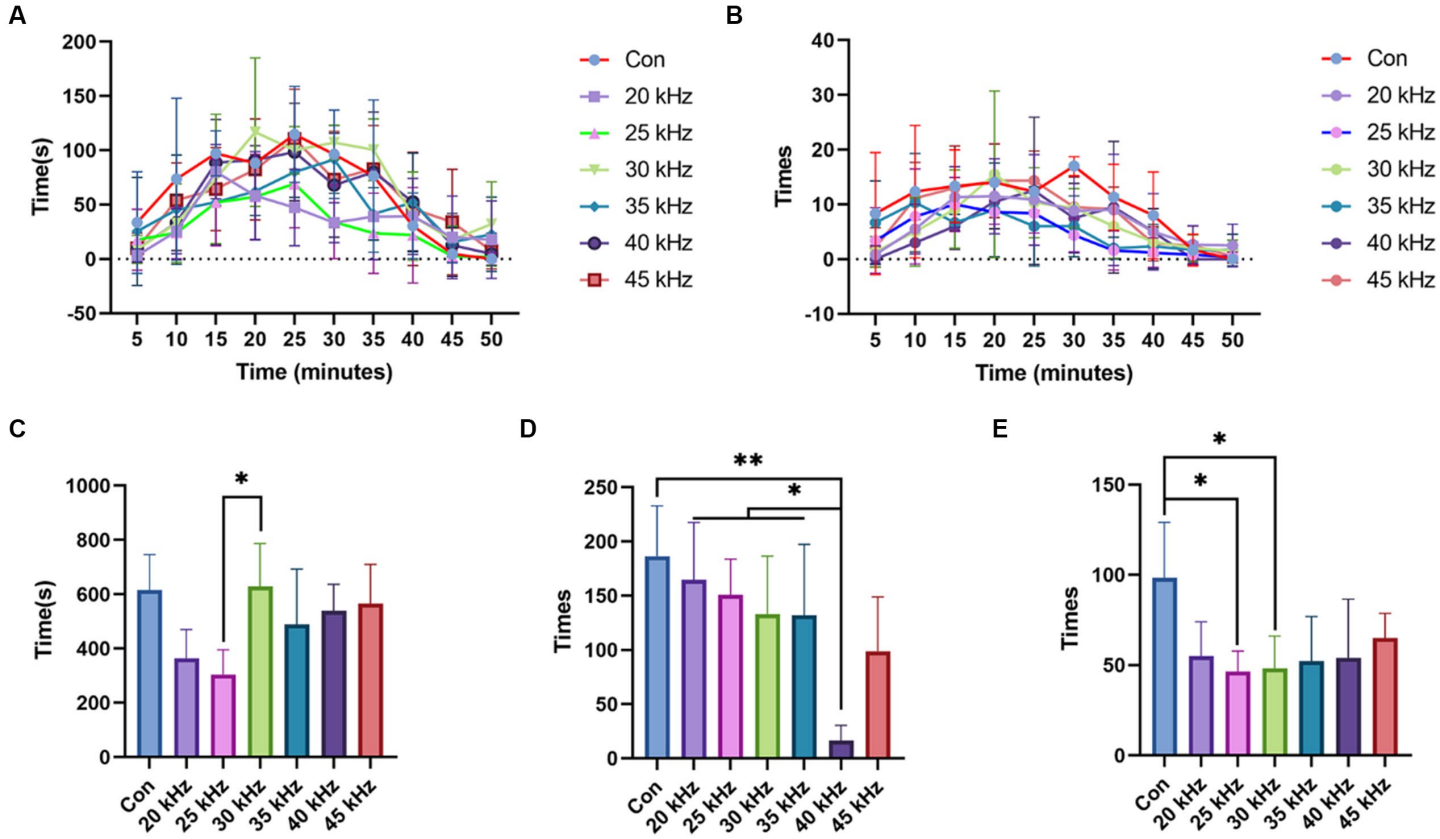
Results of the formalin test. **(A)** Time of licking the right hind paw results of formalin test with CO_2_ laser at different frequencies (*n* = 6). **(B)** Number of times of licking the right hind paw results of formalin test with CO_2_ laser at different frequencies (*n* = 6). **(C)** Total time of licking the right hind paw results of formalin test with CO_2_ laser at different frequencies (*n* = 6). **(D)** Number of total times of flinching the right hind paw results of the formalin test with CO_2_ laser at different frequencies (*n* = 6). **(E)** Number of total times of licking the right hind paw results of the formalin test with CO_2_ laser at different frequencies (*n* = 6). The data are expressed as the mean ± SD. Statistical analysis was conducted using one-way ANOVA followed by Tukey’s *post hoc* test for multiple comparisons. *p < 0.05, **p < 0.01, and ***p < 0.001.

To ascertain the optimal frequency parameter for pain relief in CO_2_ laser therapy, the formalin test was employed, providing a more comprehensive experimental approach. Following CO_2_ laser irradiation at various frequencies, the duration of right hind paw licking was recorded in 5 min intervals, with results depicted in Figure 4A. The control group clearly exhibited the highest curve, while the 25 kHz group demonstrated the lowest. During the 20 to 30 min range, the 20 kHz group displayed a brief pain-relieving interval, although this effect was short-lived and atypical. Painful behavior was most pronounced in control groups between 5 and 25 min. Intriguingly, after 25 min, some irradiated groups, including 30 kHz, 35 kHz, and 45 kHz, exhibited increased pain-related behaviors. As no other irradiated groups demonstrated worse outcomes than the control group, the abnormal response cannot be solely attributed to laser irradiation manipulation. Given its prevalence in higher frequency groups, this aberrant reaction is likely a consequence of increased irradiation intensity. Figure 4B presents the number of hind paw licking instances recorded every 5 min. The control group exhibited the highest flinching frequency throughout the observation period. Owing to the considerable behavioral statistical error, discerning the group with the lowest flinching rate is challenging. The 40 kHz group demonstrated the fewest occurrences between 5 and 15 min, while the 25 kHz group exhibited the lowest curve from 20 min onward. This suggests that the 40 kHz laser’s higher intensity facilitates faster pain relief, whereas the 25 kHz laser requires more time to achieve a similar effect. However, the 40 kHz results become less satisfactory after 20 min. Considering the licking frequency results, the 25 kHz frequency demonstrates the most effective pain relief, albeit with a longer onset time. In contrast, the 40 kHz frequency provides early pain relief but lacks consistent long-term efficacy.

Furthermore, by totaling the number of foot licking instances over the experiment, the 25 kHz group displayed the lowest count, as shown in Figure 4C. No significant differences were found between the control, 30 kHz, 35 kHz, 40 kHz, and 45 kHz groups (*p* > 0.05), each displaying similar licking frequencies. Surprisingly, the 30 kHz group exhibited a larger value than the 25 kHz group (*p* < 0.05). No significant differences were observed between the 25 kHz group and others.

The results of the formalin test with regard to the number of total times of flinching the right hind paw differed from those of the time spent licking the right hind paw (Figure 4D). Compared to the control group, a decreasing trend in the total number of right hind paw flinching was observed from 20 kHz to 40 kHz. The 40 kHz group exhibited a significant decrease in the number of flinches compared to the control group (*p* < 0.01) and 20–35 kHz groups (*p* < 0.05). Interestingly, the response pattern still conformed to a unimodal curve, with the number of flinches increasing after 40 kHz. Although the observed trend was not consistent with that observed in the licking time recording, where a frequency of 25 kHz showed the best effect for pain relief, the pain relief effect of 40 kHz irradiation was evident in the total flinching time. These findings suggest that different types of pain may correspond to varying optimal frequency parameters, as the licking and flinching behaviors represent distinct pain-related signal pathways. However, due to the large standard deviation, no significant differences were observed between the groups, except for the 40 kHz group. It is necessary to conduct further experiments with a narrower range of frequencies to determine the optimal frequency parameters for specific types of pain.

Based on the analysis of the number of total times of licking the right hind paw in the formalin test with CO_2_ laser at different frequencies (Figure 4E), our results demonstrate that 25 and 30 kHz frequencies exhibit the most effective pain relief compared to the control group (*p* < 0.05). Notably, these frequencies significantly reduced the number of times the right hind paw was licked. Conversely, the 20, 35, 40, and 45 kHz frequencies yielded lower levels of pain relief compared to the 25 kHz frequency. Overall, our findings support the hypothesis that 25 kHz may be the most suitable frequency for acute pain relief using CO_2_ laser therapy.

### 3.4. The potential biological mechanisms

**Figure 5:**
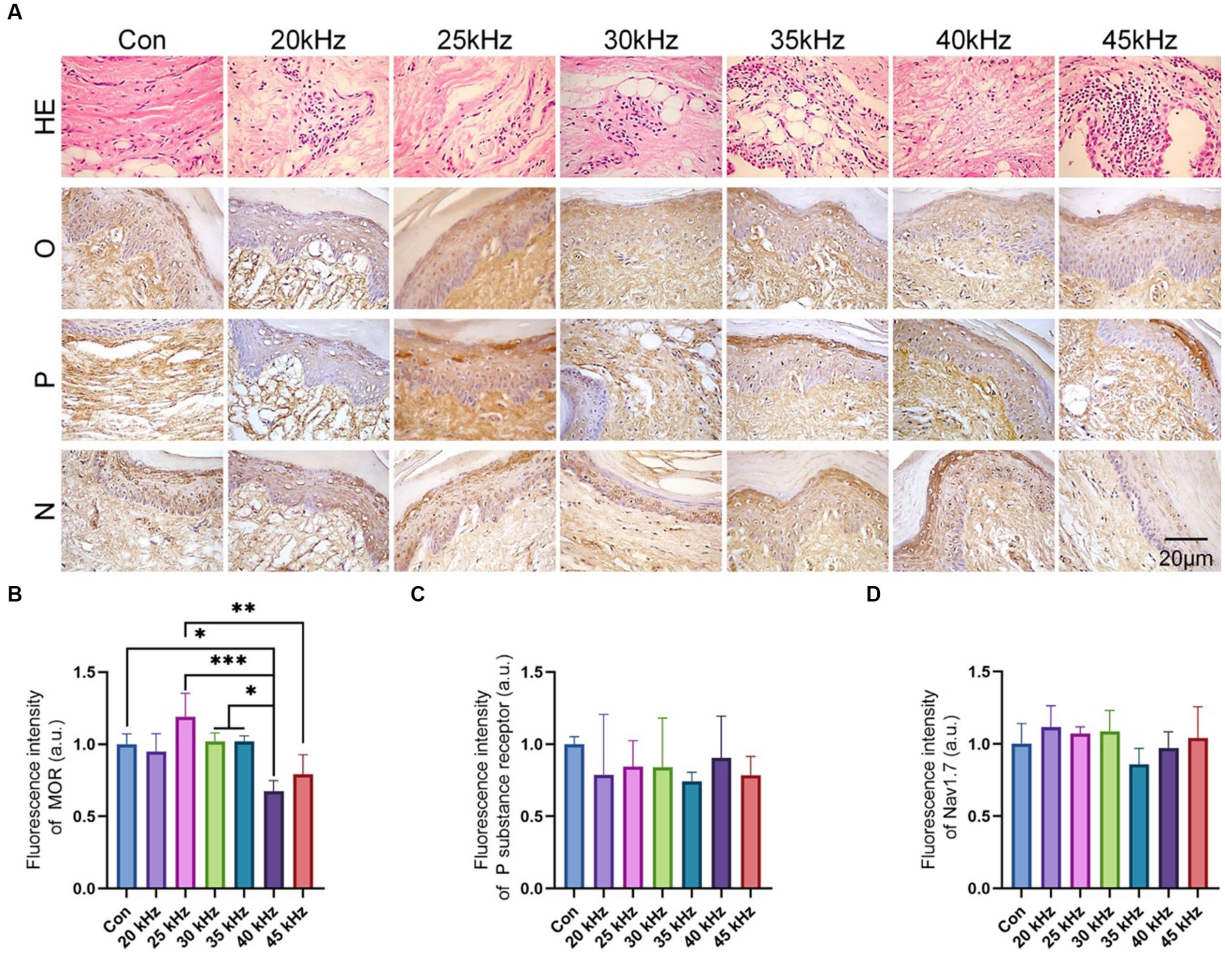
*In vivo* experimental histological staining results. **(A)** H&E and immunohistochemical staining of the hind paws after irradiation (*n* = 6). **(B)** The quantitative statistics of MOR expression (*n* = 6). **(C)** The quantitative statistics of P substance receptor expression (*n* = 6). **(D)** The quantitative statistics of Nav1.7 expression (*n* = 6). The data are expressed as the mean ± SD. Statistical analysis was conducted using one-way ANOVA followed by Tukey’s *post hoc* test for multiple comparisons. *p < 0.05, **p < 0.01, and ***p < 0.001.

In order to elucidate the underlying biological mechanism of CO_2_ laser therapy, the current study performed immunohistochemical staining of pain-related signaling pathways as shown in Figure 5. Following formalin injection, inflammation was observed in all groups (Figure 5A), with no significant difference detected among the experimental groups. Interestingly, CO_2_ laser irradiation did not mitigate or exacerbate the inflammation. To investigate the potential biological mechanism of CO_2_ laser therapy for pain relief, the study evaluated the expression levels of three commonly implicated pain-associated proteins. Notably, the immunohistochemical staining of the opioid receptor protein revealed variable intensities among different groups (Figures 5A,B), consistent with the behavioral analysis. Our results indicate that the 25 kHz group exhibited the highest level of opioid receptor protein expression compared to the 45 kHz group (*p* < 0.001) and the 50 kHz group (*p* < 0.01). It is hypothesized that the pain relief effect of CO_2_ laser therapy is mediated through the opioid receptor pathway, with different frequencies leading to varying degrees of opioid receptor silencing. Specifically, 25 kHz was identified as the most effective frequency among the tested frequencies. In contrast, the immunohistochemical staining of tissue sections (Figures 5A,C) and quantitative analysis of fluorescence intensity (Figures 5A,D) did not reveal significant differences in the expression levels of Nav1.7 protein and P substance receptor protein among all the experimental groups (*p* > 0.05). Taken together, the current results suggest that CO_2_ laser therapy is unlikely to modulate the Nav1.7 protein and P substance-related pain pathways.

## 4. Discussion

For decades, the CO_2_ laser has been widely employed as an effective surgical instrument for excision, incision, and coagulation (12, 13). However, recent investigations have shed light on the potential of the CO_2_ laser to serve as a non-ablative, non-thermal, and low-level laser for immediate pain relief in oral lesions (14, 15). Remarkably, no thermal or significant complications have been reported thus far, indicating the safety and feasibility of using the CO_2_ laser for pain management (16).

Previous studies have investigated the potential of CO_2_ laser therapy as an immediate pain-relieving treatment for various pain-related clinical conditions, including but not limited to Bechcet’s disease (BD), Aphthous stomatitis, and Pemphigus Vulgaris (17, 18). In a clinical research, Zand et al. (19) utilized the non-thermal CO_2_ laser therapy in the treatment of Behect’s disease and got promising results. Prior to irradiation, a hydrogel layer, 3–4 mm thick, was applied to the ulcer to reduce the laser intensity. The CO_2_ laser operator employed a de-focused handpiece with a power of 1 W. The therapy demonstrated significant pain relief at the lesions with no visible complications observed. In another case reported by Zand et al., a de-focused CO_2_ laser with a power of 1 W was utilized to treat genital ulcers in patients with Behcet’s disease. The study found that the application of the CO_2_ laser resulted in an immediate alleviation of pain in the affected areas (20). Furthermore, a double-blind randomized clinical trial conducted by Seyyedi et al. investigated the efficacy of both continuous and pulsed CO_2_ laser therapies in treating recurrent aphthous stomatitis (RAS). The continuous CO_2_ laser had a power of 1 W and was irradiated for 5–10 s, while the pulsed CO_2_ laser had a power of 261 W, with a pulse duration of 180 ms and a pulse interval of 40 ms. The study found that both CO_2_ laser therapies were more effective in relieving pain compared to conventional treatments for RAS (21).

Previous studies have demonstrated the immediate pain-relieving effect of CO_2_ laser therapy on oral ulcers and mucosal lesions. To ensure the safety of the operator, appropriate eyeglasses matching the CO_2_ laser should be worn during treatment (22). In addition, a transparent gel with high water content (87.5%) and a thickness of 3–4 mm is commonly applied to the ulcerated area prior to laser irradiation (23). This is done to reduce the laser intensity on the wound, as the laser strength is rapidly attenuated throughout the transparent gel. Sattayut et al. (22) conducted a detailed study using a CO_2_ laser with a power of 2 W to treat aphthous stomatitis. It demonstrated that the actual energy density throughout the transparent gel was found to be 110.67 J/cm^2^.

Numerous studies have utilized the de-focused CO_2_ laser in a continuous mode, with power ranging from 0.7–2 W and exposure times varying from 3.5 s to 10 s. The non-contact handpiece of the CO_2_ laser has been commonly employed in most investigations, with the distance between the handpiece and the wounds typically set between 5 mm and 10 mm (22). However, despite the use of CO_2_ laser therapy for pain relief, there are currently no standardized guidelines for its application. Most practitioners rely on previous experience, and there is a lack of detailed studies investigating the impact of CO_2_ laser parameters on pain relief efficacy. While output power is an important parameter, other factors such as mode, frequency, and duty cycle also play a crucial role. Thus, it is essential to comprehensively evaluate the effect of these parameters on pain-related treatments using CO_2_ laser therapy.

In this study, we carried out an investigation into the precise impact of CO_2_ laser parameters, namely duty cycle and frequency, on pain relief. To achieve this, we used CO_2_ laser in a rectangular wave mode. The duty cycle, defined as the percentage of the total time of laser exposure in a cycle, was varied in the range of 1 to 10%. It is worth noting that small duty cycles were employed in order to avoid any potential thermal effects. Our results indicate that under these duty cycles, CO_2_ laser did not induce any side effects. Immediate pain relief was observed following CO_2_ laser illumination, with different duty cycles yielding varying degrees of analgesic effects. Notably, at a duty cycle of 3%, the highest remission rates before and after irradiation were observed. Our findings provide insight into the effects of duty cycle on pain relief and underscore the importance of considering CO_2_ laser parameters beyond output power when designing pain-related treatments. Unfortunately, no significant difference was observed in behavioral measurements, likely due to a large deviation in the data. There are two possible reasons for this outcome. Firstly, it is possible that using only six animals per group was insufficient to reduce the deviation and show a statistically significant difference. Increasing the number of animals per group may provide more accurate results. Secondly, it is possible that the duty cycle is not a significant factor in pain relief. Therefore, further experiments are necessary to determine the effect of duty cycle on pain relief. In the upcoming experiments, we selected the 3% duty cycle as the most appropriate parameter for examining the effect of laser frequency.

In contrast to duty cycles, our results indicate that CO_2_ laser frequency has a significant impact on analgesic effects. We evaluated the effects of CO_2_ laser frequencies ranging from 20 kHz to 80 kHz. We observed no significant difference between the control group and the CO_2_ laser group at the highest (65 kHz, 80 kHz) and lowest (20 kHz) frequencies. However, our findings demonstrate that the frequencies of 35 kHz and 50 kHz exhibited superior analgesic effects over the other frequencies tested. These results indicate that the choice of laser frequency is an important parameter for achieving optimal analgesic outcomes. In order to further evaluate the effect of different laser frequencies on pain relief, we conducted formalin tests using frequencies ranging from 20 kHz to 50 kHz. The formalin test is a widely used method due to its ability to elicit biphasic pain-related behavior. Specifically, after a certain amount of formaldehyde injection, two consecutive response phases can be observed (24, 25). The first phase, which is related to TRPA-mediated nociceptor reaction, occurs within 0–5 min after injection, while the second phase, attributed to inflammatory or spinal sensitization, occurs within 20–60 min after injection. A quiescent interphase is observed between these two phases (26). The interphase during the formalin test is characterized by the absence of algogenic compounds, and the mechanism underlying this phenomenon remains unclear (27). In the formalin test, the response behavior of lifting, flinching, and licking the injected paw is recorded and quantified (28, 29). Prior studies have shown that an injection of formaldehyde solution in the hind paw, ranging from 19–616 mM, elicits an immediate first phase response characterized by pain-related licking behavior. Notably, the second phase of the test is only observed with formaldehyde concentrations above 39 mM, and is associated with an inflammatory or spinal sensitization response occurring 20–60 min after injection (30).

In our study, we utilized a formalin test to evaluate the analgesic effects of CO_2_ laser irradiation. Unlike the traditional formalin test, our experimental design involved administering the CO_2_ laser irradiation after formalin injection. This approach was taken because the first phase of the formalin test only lasts for 5 min, which is a relatively short duration compared to the 10 min irradiation course. Additionally, irradiation prior to formalin injection was deemed invalid as there was no pre-existing painful condition to alleviate. The formalin test is characterized by two response phases, with the second phase being primarily responsible for pain-related behavior. We used a formaldehyde solution concentration of 616 mM, which ensured that both response phases were clearly observed. Following irradiation, the behavior of the animals was recorded and analyzed.

We investigated the pain-relieving effect of CO_2_ laser in different frequencies using the formalin test. Our results showed that the CO_2_ laser frequency of 25 kHz produced the best pain-relieving effects according to the formalin test. Interestingly, we observed that under the frequency of 40 kHz, the number of flinching behaviors in the right hind paw was the lowest among all the groups. However, the different implications represented by licking and flinching behaviors in the formalin test have not been thoroughly investigated. It is therefore difficult to explain the difference in analgesic effects between 25 kHz and 40 kHz frequencies. Future studies should aim to clarify the different meanings of each behavior observed in the formalin test in order to fully understand the mechanisms underlying the pain-relieving effects of CO_2_ laser at different frequencies.

In previous studies, both continuous and pulsed low-level lasers have been repeatedly used to alleviate pain, and they have shown significant analgesic effects (31). Continuous-wave lasers do not involve the concept of frequency, whereas pulsed lasers utilize different frequencies (32). Currently, there is limited research on the influence of frequency-specific pulsed low-level lasers on analgesic effects, and the lasers used vary widely in terms of wavelength and frequency. These lasers span a range of wavelengths from 610 to 10,600 nm and frequencies from 0.01 to 100 kHz (33, 34). In one study, de Oliveira et al. employed a 9.5 kHz, 904 nm laser and demonstrated its significant relief of neuropathic pain in rats (4). In another study, Pigatto et al. used a 660 nm laser at 60 Hz, and their laser therapy significantly alleviated acute pain in mice during the formalin test, exhibiting promising therapeutic efficacy (35). It is evident that a consensus on the optimal frequency for various lasers has not yet been reached, and the optimal frequency may likely differ for lasers of different wavelengths. In this study, it is observed that frequency distinctly influences the laser’s pain-relieving effect, especially in the case of carbon dioxide lasers, where 25 kHz appears to yield the best results. It is anticipated that future research will delve further into the optimal frequency parameters for different-wavelength lasers used in analgesia.

Several studies have demonstrated the analgesic properties of CO_2_ laser therapy, but the underlying mechanism remains unclear. It is well established that long-wavelength lasers (1,200–10,600 nm) are primarily absorbed by the tissue surface, while shorter-wavelength lasers (900–1,200 nm) can penetrate deeper into the tissue (36, 37). The wavelength of CO_2_ laser, which is 10,600 nm, suggests that its energy is more likely to be absorbed at the tissue surface, potentially leading to an increase in tissue temperature. As such, the analgesic effect of CO_2_ laser is believed to be primarily related to surface analgesia. To examine the advantageous impact of CO_2_ laser on stem cells, Constantin et al. conducted a study which demonstrated that CO_2_ laser irradiation resulted in a substantial rise in the levels of intracellular reactive oxygen species (ROS) (38). The modulation of ROS levels in cells may be implicated in the observed analgesic effects following CO_2_ laser irradiation. In another study, Tsuchiya et al. (39) found the expression of glial fibrillary acidic protein (GFAP) was increased after CO_2_ laser irradiation in the treatment of orthodontic force-induced pain. The authors observed a significant increase in protein expression in the bilateral trigeminal ganglia 24 h after a 30 s irradiation of CO_2_ laser. Considering that GFAP expression has been reported to occur in dorsal root ganglia after nerve injury within 4 h, it is plausible that the analgesic effect of CO_2_ laser is related to GFAP (40).

In this study, we investigated the effect of CO_2_ laser irradiation on the expression of three common pain-related proteins, namely the opioid receptor (MOR), the Nav1.7 protein, and the P substance receptor. Our results showed no significant difference in the expression of Nav1.7 protein and the P substance receptor between the control group and the groups subjected to irradiation at various frequencies. This suggests that the analgesic effect of CO_2_ laser is not mediated via the Nav1.7 and P substance pathways. Interestingly, the expression of MOR was found to be highest in the 25 kHz irradiation group, which is consistent with the observed analgesic effect. We therefore propose that the irradiation of CO_2_ laser can induce the expression of MOR through the opioid pathway. As early as 1993, Honmura et al.’s research confirmed that low-level laser irradiation could eliminate hyperalgesia, and its analgesic effect could be completely inhibited by a certain dose of naloxone (a specific antagonist of opioid receptors), indicating that at least part of its analgesic action is induced by the release of endogenous opioid receptors (41). Later studies have also shown that low-level laser therapy can increase the release of peripheral endogenous opioid-like substances by promoting the migration of immune system cells, a process that can be antagonized by naloxone (42, 43). In this study, similar results were obtained through immunohistochemical staining, suggesting that the mechanism may be related to the opioid receptor pathway in pain relief.

In addition to opioid receptors, current research has also found that low-level laser therapy’s inhibitory effect on pain may be related to cytochrome c oxidase (44), cyclooxygenase (35), and various proinflammatory cytokines (45). The underlying molecular mechanism behind the analgesic effect of CO_2_ laser irradiation remains unclear. Specifically, it is unclear whether the laser induces changes in the conformation of the relevant proteins or promotes the expression of related genes. Therefore, further investigation is required to elucidate the molecular basis of the observed analgesic effect.

While this study provides valuable insights into the analgesic effects of CO_2_ laser therapy in rodents, caution must be exercised when extrapolating these findings directly to human research. Differences between species may affect the applicability of our data to humans. However, it is worth noting that previous clinical studies have indeed demonstrated significant pain-inhibiting effects of low-level laser therapy in humans (46). Furthermore, summarizing previous research reveals a certain degree of similarity in laser parameters used for rodents and humans to achieve pain inhibition; the laser wavelength, power, and energy used are almost the same (34, 47). Therefore, we are optimistic about translating the results of this study into human research. In future research, we look forward to more in-depth investigations into the mechanisms of CO_2_ laser therapy, with a particular focus on elucidating the potential pathways and biological mechanisms involved. Additionally, we anticipate more clinical studies to explore the application of CO_2_ laser therapy in pain relief in humans. By combining these approaches, we aim to bridge the gap between preclinical and clinical research, providing a more comprehensive understanding of the therapeutic potential of CO_2_ laser therapy in pain management.

## 5. Conclusion

In summary, this study provides a detailed evaluation of the analgesic effects of CO_2_ laser therapy under various parameters, specifically the duty cycle and frequency. Our findings reveal that the degree of CO_2_ laser significantly influences its acute pain-relieving effect. Using the hot plate and formalin tests, we determined that the most suitable duty cycle is 3% and the most suitable frequency is 25 kHz. However, under a frequency of 40 kHz, the CO_2_ laser exhibited a better effect than at 25 kHz in flinching of the right hind paw during the formalin test, suggesting different mechanisms at different frequencies. Furthermore, we employed immunohistochemistry to investigate the potential mechanisms of CO_2_ laser therapy, examining three common pain-related protein signal pathways. Our results suggest that the analgesic effect of CO_2_ laser therapy is likely associated with the opioid receptor signal pathway. Taken together, our study provides an elaborate explanation of the influence of CO_2_ laser parameters on its analgesic effect and its potential mechanism of action.

## Data availability statement

The original contributions presented in the study are included in the article/supplementary material, further inquiries can be directed to the corresponding author.

## Ethics statement

The animal experimental protocols were approved by the Animal Care and Use Ethics Committee of Jilin University. The study was conducted in accordance with the local legislation and institutional requirements.

## Author contributions

XW: Conceptualization, Formal analysis, Investigation, Methodology, Writing – original draft, Writing – review & editing. JL: Formal analysis, Investigation, Methodology, Writing – original draft. ZW: Formal analysis, Methodology, Writing – original draft. CG: Formal analysis, Methodology, Writing – original draft. HoL: Methodology, Resources, Writing – original draft. SF: Investigation, Methodology, Writing – original draft. HeL: Investigation, Methodology, Writing – original draft. XG: Resources, Supervision, Writing – original draft. DZ: Methodology, Resources, Writing – original draft. LZ: Writing – original draft. HJ: Conceptualization, Formal analysis, Funding acquisition, Methodology, Project administration, Writing – review & editing. JW: Funding acquisition, Resources, Supervision, Writing – review & editing.
